# Safety and efficacy of *N*-acetylmannosamine (ManNAc) in patients with GNE myopathy: an open-label phase 2 study

**DOI:** 10.1038/s41436-021-01259-x

**Published:** 2021-07-13

**Authors:** Nuria Carrillo, May C. Malicdan, Petcharat Leoyklang, Joseph A. Shrader, Galen Joe, Christina Slota, John Perreault, John D. Heiss, Bradley Class, Chia-Ying Liu, Kennan Bradley, Colleen Jodarski, Carla Ciccone, Claire Driscoll, Rebecca Parks, Scott Van Wart, Levent Bayman, Christopher S. Coffey, Melanie Quintana, Scott M. Berry, Marjan Huizing, William A. Gahl

**Affiliations:** 1 National Human Genome Research Institute, National Institutes of Health, Bethesda, MD USA; 2 Therapeutics for Rare and Neglected Diseases, National Institutes of Health, Bethesda, MD USA; 3 Department of Rehabilitation Medicine, NIH Clinical Center, National Institutes of Health, Bethesda, MD USA; 4 National Institute of Neurological Disorders and Stroke, National Institutes of Health, Bethesda, MD USA; 5 Radiology and Imaging Sciences, NIH Clinical Center, National Institutes of Health, Bethesda, MD USA; 6 Enhanced Pharmacodynamics LLC, Buffalo, NY USA; 7 Clinical Trials Statistical and Data Management Center, University of Iowa, Iowa City, IA USA; 8 Berry Consultants LLC, Austin, TX USA

## Abstract

**Purpose:**

To evaluate the safety and efficacy of *N*-acetylmannosamine (ManNAc) in GNE myopathy, a genetic muscle disease caused by deficiency of the rate-limiting enzyme in *N*-acetylneuraminic acid (Neu5Ac) biosynthesis.

**Methods:**

We conducted an open-label, phase 2, single-center (NIH, USA) study to evaluate oral ManNAc in 12 patients with GNE myopathy (ClinicalTrials.gov NCT02346461). Primary endpoints were safety and biochemical efficacy as determined by change in plasma Neu5Ac and sarcolemmal sialylation. Clinical efficacy was evaluated using secondary outcome measures as part of study extensions, and a disease progression model (GNE-DPM) was tested as an efficacy analysis method.

**Results:**

Most drug-related adverse events were gastrointestinal, and there were no serious adverse events. Increased plasma Neu5Ac (+2,159 nmol/L, *p* < 0.0001) and sarcolemmal sialylation (*p* = 0.0090) were observed at day 90 compared to baseline. A slower rate of decline was observed for upper extremity strength (*p* = 0.0139), lower extremity strength (*p* = 0.0006), and the Adult Myopathy Assessment Tool (*p* = 0.0453), compared to natural history. Decreased disease progression was estimated at 12 (γ = 0.61 [95% CI: 0.09, 1.27]) and 18 months (γ = 0.55 [95% CI: 0.12, 1.02]) using the GNE-DPM.

**Conclusion:**

ManNAc showed long-term safety, biochemical efficacy consistent with the intended mechanism of action, and preliminary evidence clinical efficacy in patients with GNE myopathy.

## INTRODUCTION

GNE myopathy (OMIM 605820) is a rare autosomal recessive inborn error of sialic acid biosynthesis that manifests as progressive skeletal muscle atrophy in young adults [1]. The disease slowly progresses to eventually affect skeletal muscles throughout the body, significantly impairing physical function and quality of life and leading to dependent care for activities of daily living (ADLs) [2, 3]. GNE myopathy is caused by variants in the UDP-*N*-acetylglucosamine (UDP-GlcNAc) 2-epimerase/*N*-acetylmannosamine (ManNAc) kinase (*GNE*) gene that result in decreased activity of this bifunctional enzyme responsible for initiating and regulating the intracellular biosynthesis of *N*-acetylneuraminic acid (Neu5Ac, sialic acid) [4–7]. Decreased biosynthesis of Neu5Ac and the subsequent hyposialylation of skeletal muscle glycoproteins are considered to play a critical role in the disease [8–12], although the precise mechanism by which these lead to muscle atrophy and weakness continues to be studied. There is no approved treatment for this debilitating disease.

ManNAc, an uncharged monosaccharide and the first committed precursor in the Neu5Ac biosynthesis pathway, is an orphan drug in development for the treatment of GNE myopathy. Preclinical studies have shown that ManNAc increases Neu5Ac biosynthesis and sialylation in various unaffected and disease models [7, 9, 13–19]. Oral administration of ManNAc increased Neu5Ac biosynthesis, improved muscle sialylation, and prevented skeletal muscle deterioration in a mouse model that recapitulates the human disease [17]. A first-in-human, randomized, placebo-controlled, double-blind study (NCT01634750) showed that single doses of oral ManNAc were safe and led to a sustained increase in plasma Neu5Ac levels in patients with GNE myopathy [20]. Here, we report the results of an open-label phase 2 study that evaluated oral ManNAc in adults with GNE myopathy. We provide evidence of the long-term safety and biochemical efficacy of ManNAc as evidenced by plasma Neu5Ac concentrations and sarcolemmal sialylation. Additionally, secondary outcome measures of strength and function were evaluated for clinical efficacy in patients with GNE myopathy.

## MATERIALS AND METHODS

This was an open-label, phase 2, single-center study conducted at the NIH Clinical Center (Bethesda, MD, USA) under National Institutes of Health (NIH) study 15-HG-0068 “An Open-Label Phase 2 Study of ManNAc in Patients with GNE Myopathy.” The protocol was approved by the National Human Genome Research Institute (NHGRI) Institutional Review Board (IRB). All patients gave written, informed consent and received ManNAc under FDA Investigational New Drug Application 078091. The study is registered at ClinicalTrials.gov, identifier NCT02346461.

Primary endpoint follow-up visit occurred on day 90. The protocol was extended from 90 days to 6 months as supported by clinical safety and long-term animal toxicology studies. As clinical safety accumulated, the study was extended with follow-up visits at 12, 18, 24, and 30 months for collection of long-term safety and clinical outcome measures.

### Patients

Eligible patients were of either sex between 18 to 60 years of age, with a diagnosis of GNE myopathy based upon consistent clinical findings and identification of biallelic pathogenic variants in *GNE*, body mass index (BMI) between 18 and 30 kg/m^2^, body weight >50 kg, and muscle strength at baseline between 20% and 75% of predicted for ≥1 of the following: ankle dorsiflexion, knee flexion, hip extension, grip, elbow flexion or shoulder abduction. Patients were ineligible if they had received ManNAc, sialic acid, intravenous immunoglobulin (IVIG), or other compounds containing sialic acid within 90 days before the baseline visit or had a history of persistent diarrhea or malabsorption.

### Intervention

N-acetyl-D-mannosamine monohydrate (ManNAc) was manufactured by New Zealand Pharmaceuticals Ltd (Palmerston North, New Zealand). Patients were sequentially assigned to receive either 3 g or 6 g twice daily (BID) of oral ManNAc for 7 days. After day 7, all patients received doses of 6 g BID (12 g/day) supported by safety data of the 6 g BID cohort and long-term toxicology studies performed in rats (26 weeks) and dogs (39 weeks).

### Outcomes

Safety assessments collected throughout the study included adverse events, physical and neurological examinations, vital signs, and clinical laboratory tests (serum chemistry, hematology, and urinalysis). Adverse events were classified based on the Common Terminology Criteria for Adverse Events (CTCAE) v4.0.

Biochemical efficacy was evaluated by plasma Neu5Ac, intracellular CMP-Neu5Ac and sarcolemmal sialylation. Plasma ManNAc and Neu5Ac concentrations were determined by Alliance Pharma, Inc. (Malvern, PA) using a validated high-performance liquid chromatography with tandem mass spectrometry (HPLC-MS/MS) method, as previously reported [21]. Intracellular concentrations of CMP-Neu5Ac were measured on white blood cell (WBC) pellets by LC/MS-MS, as previously reported [22]. Change in sarcolemmal sialylation from baseline to day 90 was evaluated by a blinded evaluator on muscle biopsies using a quantitative lectin fluorescence method to determine the *Sambucus nigra* agglutinin (SNA), a lectin that predominantly recognizes terminal α2,6-linked Neu5Ac (Neu5Acα2,6 Galβ), colocalized with the sarcolemmal protein Caveolin-3 (Cav-3), as previously described [23]. Muscle biopsies were obtained from biceps brachii and a lower extremity muscle at baseline and at day 90 for a total of up to four specimens (two pairs) per patient. To sample actively diseased muscles, the lower extremity biopsy site was selected in each patient by identifying muscle regions with minimal fatty replacement and short tau inversion recovery (STIR) hyperintensity on muscle MRI (3-T whole-body MRI system, Verio, Siemens Medical Systems, Erlangen, Germany), as previously described [24]. To ensure that differences in sialylation were due to the intervention rather than the variability of sialylation among different muscles groups, day 90 samples were obtained immediately adjacent to the baseline biopsy sites by extending the previous incision. Cryosections were co-stained with SNA (Vector Laboratories, CA) and antibodies to the sarcolemmal protein Cav-3 (R&D Systems, Minneapolis, MN). Sectioning, fluorescence staining, image acquisition, and determination of total membrane length per cryosection were performed as previously reported [23]. Cryosections were reviewed to ensure good quality and excluded from analysis if unreliable for quantification. SNA and Cav-3 fluorescence quantifications were performed by a blinded evaluator (Definiens, Cambridge, MA) on whole slide images using Definiens Architect Software with specialized algorithms. Because muscle sections varied in size, parameters were calculated per total membrane length in each section.

Secondary clinical outcomes included change from baseline of measures of muscle strength, function and patient-reported outcomes, which were collected every 6 months for up to 30 months. Quantitative muscle strength was measured by an experienced physical therapist using a fixed frame dynamometer (QMA, Aeverl Medical, Gainesville GA, USA) to record maximal voluntary isometric contraction in kg and expressed as percent of predicted strength for age, sex, height, and weight [25]. Upper extremity composite strength was calculated as the sum of the bilateral grip, wrist extension, elbow flexion, elbow extension, and shoulder abduction. Lower extremity strength as the sum of the bilateral ankle dorsiflexion, knee flexion, knee extension, hip extension, and hip abduction. Measures of function included the 6-minute walk test (6MWT), and the Adult Myopathy Assessment Tool (AMAT), a valid and reliable performance test that assesses physical function and endurance [26, 27]. Patient-reported outcomes included the Human Activity Profile (HAP) and the Inclusion Body Myositis Functional Rating Scale (IBMFRS) to evaluate physical activity levels and ADLs, respectively [3].

### Statistical analyses

The sample size was selected to evaluate safety, pharmacokinetic analyses, and biochemical efficacy. Statistical analyses for safety and biochemical efficacy were performed in SAS 9.4 (SAS Institute Inc., 2012, Cary, NC, USA) by the Clinical Trials Statistical and Data Management Center at the University of Iowa. For biochemical efficacy, a mixed effects model with repeated measures was used to estimate the change at day 90 from baseline for SNA fluorescence intensity. Measures from a single subject/muscle were considered repeated measures. Only muscle biopsy pairs that had reliable quality and fluorescent staining for both timepoints from the same subject/muscle were included in the analyses. Pharmacokinetic parameters were estimated using noncompartmental analysis by KinderPharm, LLC (Exton, PA) with validated Phoenix WinNonlin® v8.0 software (Pharsight, Cary, NC, USA). A population pharmacokinetic/pharmacodynamic model was used to simultaneously characterize plasma ManNAc and Neu5Ac following oral administration of ManNAc to patients with GNE myopathy, as previously described [28].

To evaluate the effect on disease progression, the percent of predicted muscle strength of six muscle groups was analyzed by incorporating a treatment effect parameter, gamma (γ), into a Bayesian model of disease progression, the GNE Myopathy Disease Progression Model (GNE-DPM), as previously described [29]. The posterior mean and 95% confidence interval of the treatment effect parameter (**γ**) (γ = 1: no treatment effect, γ = 0: stop in disease progression), and the posterior probability of treatment effect [Pr(γ < 1)] were estimated. The decline for various clinical measures was estimated by fitting a linear model in the change from baseline as a function of length of follow-up incorporating all datapoints collected with no imputation for patients that did not participate in all timepoints and compared to our previously published natural history estimates, as previously described [29], using least squares regression.

## RESULTS

**Fig. 1:**
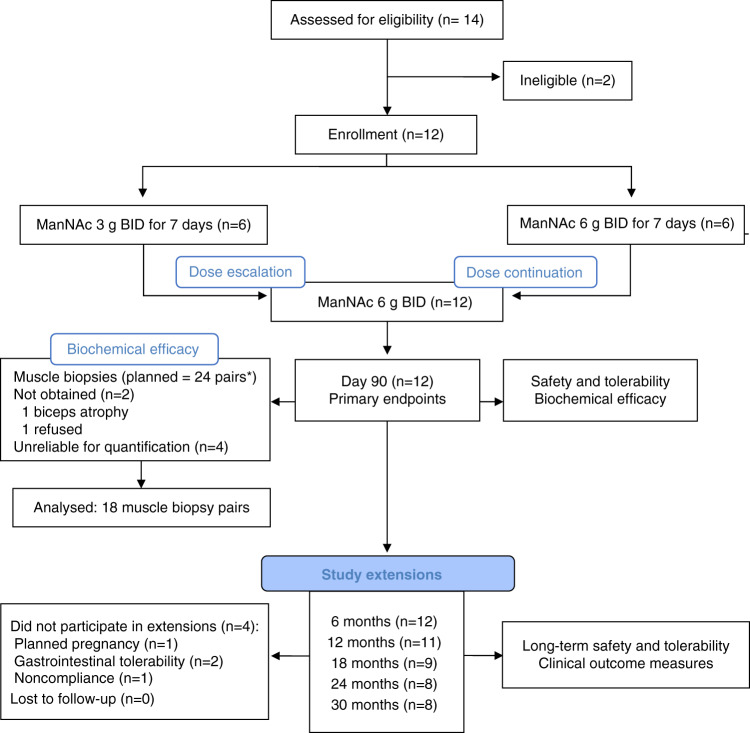
Flow diagram. *Of the 24 planned muscle biopsy pairs, 18 pairs were included in the analysis. BID twice daily.

Demographics and baseline characteristics are summarized in Table 1. In all patients, the diagnosis of GNE myopathy was confirmed by identification of biallelic pathogenic variants. Patients were distributed evenly for sex and had a mean age of 38 years at baseline. The cohort represented a wide spectrum of disease severity as determined by years from the onset of disease to enrollment (mean: 12 years, range 1–32), ambulatory device use and clinical outcome measures at baseline (Table 1). All patients completed primary endpoint follow-up visit at day 90. Patients were assessed for long-term safety and secondary clinical outcomes as part of study extensions at 6 (*n* = 12), 12 (*n* = 11), 18 (*n* = 9), 24 (*n* = 8), and 30 months (*n* = 8) (Fig. 1).

**Table 1:** Demographics and baseline characteristics.

	Patients (*n* = 12)
Age (years)	38 (25–55)
Female	6 (50%)
Race	
White	8 (67%)
Asian	4 (33%)
Age at disease onset (years)	26 (20–31)
Disease onset to enrollment (years)	12 (1–32)
Weight (kg)	78.7 (19.3)
BMI (kg/m^2^)	25.9 (3.6)
*GNE* domain affected	
Epimerase/kinase	10 (83%)
Kinase/kinase	2 (17%)
Use of any assistive ambulatory device	8 (67%)
Use of wheelchair	3 (25%)
Plasma ManNAc (nmol/L)^a^	402 (140)
Plasma Neu5Ac (nmol/L)^b^	437 (139)
QMA upper extremity strength (kg)	111 (1.5–294)
QMA lower extremity strength (kg)	165 (55.6–292)
6MWT distance (meters)^c^	423 (276–565)
AMAT total score (range 0–45)	32.17 (4–43)
HAP adjusted activity score (range 0–94)	59.17 (7–94)
HAP maximum activity score (range 0–94)	73.92 (15–94)
IBMFRS total score (range 0–40)	31.75 (10–40)

Data are *n* (%), mean (SD or range).

*6MWT* six-minute walk test, *AMAT* Adult Myopathy Assessment Tool, *BMI* body mass index, *HAP* human activity profile, *IBMFRS* Inclusion Body Myositis Functional Rating Scale, *QMA* quantitative muscle assessment.

^a^SI conversion for plasma concentrations of ManNAc (ng/ml)*4.517 = nmol/L.

^b^SI conversion for plasma concentrations of Neu5Ac (ng/ml)*3.237 = nmol/L.

^c^SI One nonambulatory patient was unable to perform the test.

Long-term safety was monitored for up to 30 months of 6 g oral ManNAc BID administration (12 g/day). There were no major safety concerns or serious adverse events. All patients experienced adverse events during the study, with most classified as grade 1 (80%) or grade 2 (14%) (Supplemental Table 1). All grade 3 adverse events (*n* = 9) were considered by investigators to be unrelated or unlikely related to study drug, except one event of grade 3 hypertriglyceridemia seen at day 895 in a patient who had baseline hypertriglyceridemia, a positive family history, and weight gain during the study. Hypertriglyceridemia resolved without intervention in three other patients. Mild transaminase elevations and hypercholesterolemia were documented in 75% and 25% of patients, respectively, but were not considered clinically significant. There were no clinically significant vital sign abnormalities. Gastrointestinal adverse events were common, including flatulence (67%), bloating (42%), and diarrhea (25%). Three patients had flatulence almost daily without associated abdominal pain or vomiting; two of them chose not to participate in study extensions due to this tolerability issue (Fig. 1). The gastrointestinal symptoms typically appeared within hours of ManNAc administration and were more commonly associated with the morning dose or taking ManNAc on an empty stomach. The majority of patients who continued to receive ManNAc as part of the study extensions noted improved gastrointestinal tolerability over time. The gastrointestinal tolerability issues were likely due to incomplete absorption of ManNAc at doses of 6 g BID. Doses of 4 grams three times daily (TID; same 12 g/day dose) led to an increased extent of absorption of ManNAc compared to the 6 g BID regimen, as shown by PK studies performed at the 30-month visit (Supplemental Table 2).

**Fig. 2:**
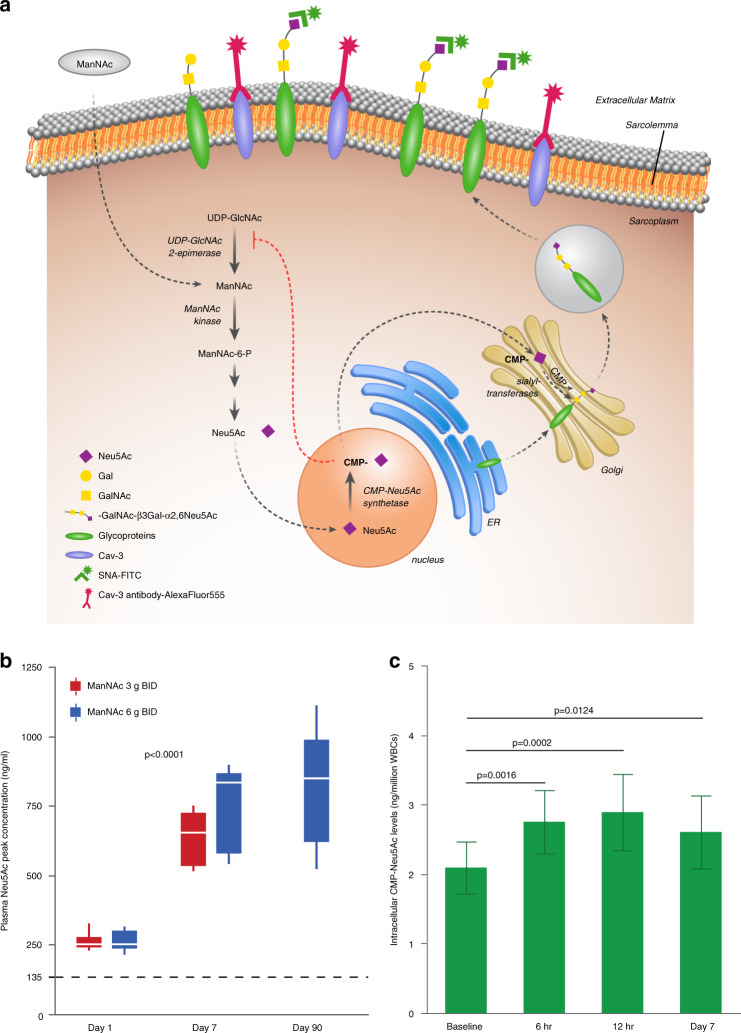
Neu5Ac production. (**a**) Decreased enzymatic activity of UDP-*N*-acetylglucosamine 2-epimerase/*N*-acetylmannosamine kinase (GNE) results in impaired Neu5Ac production and glycoprotein sialylation. The rate-limiting step in the pathway is catalyzed by *UDP-GlcNAc 2-epimerase*. ManNAc is phosphorylated by *ManNAc kinase*. Neu5Ac is activated in the cell nucleus to CMP-Neu5Ac, which acts as the donor of Neu5Ac in the reactions catalyzed by sialyltransferases to sialylate nascent glycoproteins in the Golgi. Sialylated glycoproteins are abundant on plasma membranes where they mediate several biological processes such as cellular adhesion, cell interactions, and signal transduction. FITC-labeled SNA lectin (green), which predominantly binds to terminal α2,6-linked Neu5Ac (Neu5Acα2,6Galβ), and antibodies against the sarcolemmal residence protein Caveolin-3 (Cav-3), are shown. *Figure courtesy of Julia Fekecs*. (**b**) Plasma peak concentrations of Neu5Ac by timepoint and dose. The dotted line denotes mean plasma concentration at baseline. To obtain SI units, multiply plasma Neu5Ac in ng/ml by 3.237 to obtain the concentration in nmol/L. (**c**) Intracellular CMP-Neu5Ac concentrations (mean, SD) measured by liquid chromatography/tandem mass spectrometry (LC/MS-MS) in white blood cells (WBCs) at baseline, 6 and 12 hours after initial dosing, and trough on day 7.

ManNAc was rapidly absorbed and exhibited a high apparent volume of distribution, consistent with extensive distributions to tissues. Plasma Neu5Ac concentrations increased following the ManNAc administration with mean peak plasma concentrations observed 6–8 hours after oral dosing, consistent with previous results. However, plasma Neu5Ac concentrations increased further with repeated ManNAc administration, reaching steady-state levels on day 7, which were significantly higher than baseline values (*p* < 0.0001) (Fig. 2b, Supplemental Table 2), including in patients homozygous for ManNAc kinase enzymatic domain pathogenic variants (Supplemental Fig. 1A). The dose–response relationship between ManNAc administration and plasma Neu5Ac evaluated using the population PK/PD model, confirmed an increase in the conversion efficiency of ManNAc to Neu5Ac with repeated dosing (Supplemental Fig. 1B). Intracellular concentrations of CMP-Neu5Ac in WBCs were higher at 6 hours postdose (*p* = 0.0016) and 12 hours postdose (*p* = 0.0002) compared to baseline and trough levels remained elevated on day 7 (*p* = 0.0124) (Fig. 2c), consistent with intracellular restoration of the Neu5Ac biosynthesis pathway. At day 90, the mean plasma Neu5Ac concentration was 802 ng/ml (2,596 nmol/L) at ManNAc 6 g BID (*n* = 12), compared to 135 ng/ml (437 nmol/L) at baseline (Supplemental Table 2).

**Fig. 3:**
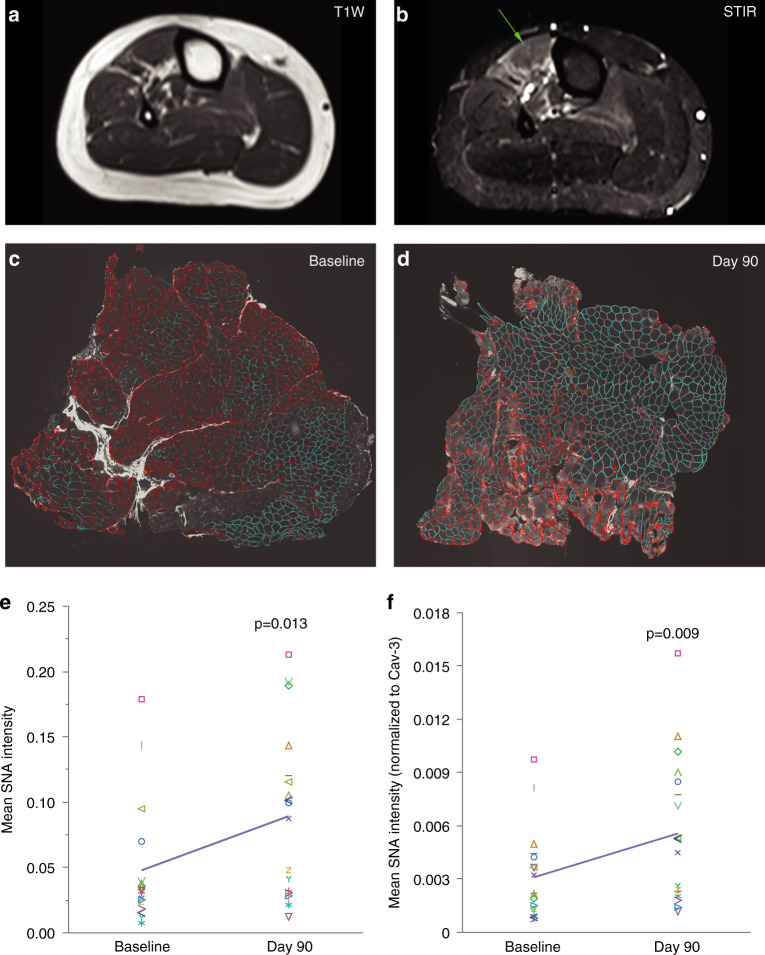
Sarcolemmal sialylation. (**a**, **b**) Selection of muscle biopsy sites by muscle magnetic resonance image (MRI) in lower extremity muscles. Muscle regions with active disease (arrow) were identified by (**a**) absence of significant fat replacement on T1-weighted (T1W) and (**b**) short tau inversion recovery (STIR) hyperintensity (arrow). (**c**, **d**) Staining of muscle cryosections with the sarcolemmal marker Caveolin-3 (Cav-3, red) and the SNA lectin that recognizes sialylation (green-blue) at (**c**) baseline and (**d**) following 90 days of daily ManNAc administration. (**e**, **f**) Sarcolemmal sialylation increased at day 90 compared to baseline as measured by (**e**) mean and (**f**) normalized SNA intensities. Each symbol represents a muscle biopsy pair at baseline and day 90 (*n* = 18), and the line connects the means of both timepoints.

To assess the biochemical efficacy of ManNAc in the target muscle tissue, sarcolemmal sialylation was evaluated in matched pairs of actively diseased muscles of the upper and lower extremities (Fig. 3a–b) using a quantitative SNA lectin fluorescence method performed by a blinded evaluator (Fig. 3c–d). Of the 24 potential muscle biopsy pairs, two were not obtained and four (8%) cryosections were determined to be unreliable for quantification (Fig. 1). Evaluation of the remaining 18 muscle biopsy pairs showed increased sarcolemmal sialylation at day 90 compared to baseline as measured by mean SNA intensity (*p* = 0.013), and mean SNA intensity normalized to Cav-3 (*p* = 0.009) (Fig. 3e–f).

**Fig. 4:**
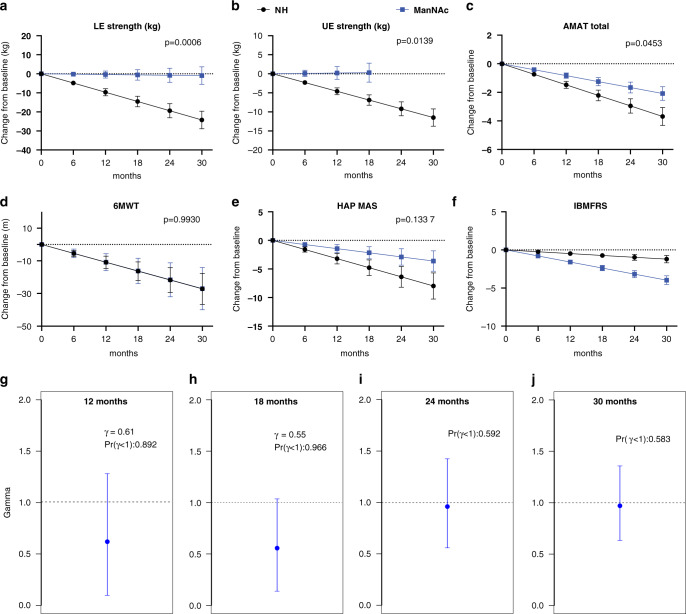
Clinical efficacy of ManNAc. (**a**–**f**) Clinical efficacy evaluated as the estimated decline for various exploratory clinical efficacy measures in patients with GNE myopathy treated with ManNAc (blue) compared with previously reported natural history (NH) estimates (black), including for (**a**) lower extremity (LE) strength, (**b**) upper extremity (UE) strength, (**c**) Adult Myopathy Assessment Tool (AMAT) total score, (**d**) 6-minute walk test (6MWT), (**e**) human activity profile maximum activity score (HAP MAS) and (**f**) Inclusion Body Myositis Functional Rating Scale (IBMFRS). (**g**–**j**) Posterior distribution of treatment effect as estimated by the GNE Myopathy Disease Progression Model (GNE-DPM) at (**g**) 12 months, (**h**) 18 months, (**i**) 24 months, and (**j**) 30 months, showing the posterior mean (blue marker) with 95% confidence intervals (blue line) of the treatment effect parameter (gamma, γ), and the posterior probability that ManNAc decreased disease progression [Pr(γ < 1)].

Secondary clinical outcome measures were assessed in patients participating in study extensions, although the trial was not designed or powered to evaluate clinical efficacy. The decline of various clinical outcome measures was compared to previous natural history estimates [24]. There was a significantly slower rate of decline under treatment compared to natural history for upper extremity strength (*p* = 0.0139), lower extremity strength (*p* = 0.0006), and AMAT total score (*p* = 0.0453), but not for the 6MWT, the HAP, or the IBMFRS (Fig. 4a–f and Supplemental Table 3). A decrease in disease progression at 12 and 18 months was estimated using the GNE-DPM with mean estimates for gamma (γ) of 0.61 (95% CI: 0.09, 1.27) at 12 months and 0.55 (95% CI: 0.12, 1.02) at 18 months, which translate to an estimated reduction of 39% and 45% in the rate of disease progression at 12 and 18 months, respectively. The posterior probabilities that the treatment decreased the rate of disease progression [Pr(γ < 1)] were estimated to be 0.89 and 0.96 at 12 and 18 months, respectively (Fig. 4g, h). The results obtained at 24 and 30 months (*n* = 8) had posterior probabilities of 0.58 and 0.59, respectively (Fig. 4i, j).

## DISCUSSION

GNE myopathy is a genetic muscle disease caused by decreased activity of the rate-limiting bifunctional enzyme in the intracellular pathway of Neu5Ac biosynthesis, resulting in impaired Neu5Ac production and cell surface hyposialylation [5, 7, 9, 14, 30]. The disease is characterized by progressive muscle atrophy and weakness, sequentially involving skeletal muscles throughout the body [24, 29]. Ultimately, patients require a wheelchair for mobility and caregiver assistance to perform activities of daily living [2]. ManNAc is a neutral amino sugar and the first committed precursor in the intracellular Neu5Ac biosynthesis pathway (Fig. 2a). Abundant experimental evidence shows that ManNAc is efficiently incorporated into the pathway to produce Neu5Ac and CMP-Neu5Ac in a dose-dependent manner, and increases sialylation of cell surface glycoconjugates [7, 9, 13–19]. ManNAc administration improved hyposialylation in two independent preclinical studies [17, 19], and prevented the muscle phenotype in a mouse model of the disease [17]. Clinical evidence from the first-in-human study of ManNAc showed a sustained increase in plasma Neu5Ac after a single dose of ManNAc [20]. Results from this study showed that oral ManNAc is safe, improved Neu5Ac biosynthesis and sarcolemmal sialylation, and may reduce disease progression in patients with GNE myopathy.

The long-term safety of ManNAc was monitored for up to 30 months, showing no major safety concerns. Hypertriglyceridemia, which was also observed in animal toxicology studies, should be considered a potential risk, particularly in patients with familial and environmental predispositions. Gastrointestinal adverse events were common and likely due to incomplete absorption of ManNAc at doses of 6 g BID. We showed that dividing the same daily dose in 4 g TID increases ManNAc absorption, with the potential to improve gastrointestinal tolerability.

The biochemical efficacy findings in this study show increased intracellular biosynthesis of Neu5Ac with the administration of ManNAc in patients with GNE myopathy. The high apparent volume of distribution for ManNAc is consistent with previous evidence that ManNAc distributes extensively to tissues including muscle [31], and crosses the plasma membrane readily [13]. Once intracellular, ManNAc has been shown to serve as a precursor for sialic acid biosynthesis, bypassing the rate-limiting feedback inhibition enzymatic step catalyzed by UDP-GlcNAc 2-epimerase [7, 13, 14]. In patients with GNE myopathy, genetic alterations in either the epimerase or kinase enzymatic domains of *GNE* decrease both enzyme activities independently of the domain affected, as it is typical for bifunctional enzymes [5, 6, 14]. Therefore, the increase in plasma Neu5Ac after ManNAc administration to patients with GNE myopathy, including those with kinase domain pathogenic variants, which was also observed in our previous phase 1 study [20], shows that ManNAc can be successfully incorporated into the Neu5Ac biosynthesis pathway despite decreased ManNAc kinase activity. The mechanism by which ManNAc can be incorporated into the pathway in patients with GNE myopathy could involve residual ManNAc kinase activity or phosphorylation of ManNAc by other kinases such as GlcNAc kinase [14, 18]. In fact, the peak plasma Neu5Ac concentrations after multiple doses of ManNAc in this study were higher than previously reported after the administration of the same dose of extended-release sialic acid (Ace-ER) to patients with GNE myopathy [32, 33]. Furthermore, we found an increase in Neu5Ac production with repeated ManNAc administration, which could be consistent with increased efficiency in the conversion of ManNAc to Neu5Ac with treatment duration. ManNAc may stabilize or improve the residual activity of this bifunctional enzyme by substrate chaperoning. In fact, one study showed that ManNAc increased the expression and the activity of UDP-GlcNAc 2-epimerase in muscle tissue of affected mice [16]. The increase in intracellular CMP-Neu5Ac, which is the end-product of the pathway and the donor of Neu5Ac to nascent glycoconjugates in the Golgi, provided further evidence of restoration of the intracellular pathway.

There is growing evidence linking inadequate sialylation of sarcolemmal glycans to the muscle pathology in GNE myopathy[9–12, 17, 19, 30]. In this study, we observed an increase in sarcolemmal sialylation after 90 days of ManNAc administration compared to baseline in patients with GNE myopathy. Historically, muscle sialylation had been assessed by measuring total bound Neu5Ac in muscle tissue; however, this methodology does not allow for the accurate comparison of Neu5Ac attached to sarcolemmal glycoproteins. The method used in this study quantifies SNA colocalized with the sarcolemmal protein Cav-3, allowing a more accurate determination of sarcolemmal sialylation [23]. Several variables were controlled to ensure that changes in sialylation were due to treatment and not to other factors. To ensure that changes in sialylation were not due to variability of sialylation among muscle groups, muscle biopsy pairs were obtained from the same muscle region. Since this was an open-label study, sarcolemmal sialylation analysis was performed by a blinded evaluator using a quantitative imaging method, as previously described. The increase in sarcolemmal sialylation provides evidence that ManNAc can reach the sarcoplasm, restore the intracellular Neu5Ac pathway, and increase glycoprotein sialylation in skeletal muscles (Fig. 2a). It has been shown that the removal of sarcolemmal sialic acid residues results in altered function and integrity of the sarcolemma [34]. Sialylated sarcolemmal glycoproteins include α-dystroglycan, neural cell adhesion molecule (NCAM), neprilysin, insulin-like growth factor 1 (IGFR1), tumor necrosis factor receptor 1 (TNFR1), and β_1_-integrin, which play roles in skeletal muscle cell signaling pathways, cytoskeleton and extracellular matrix interactions, muscle regeneration, and response to stress and mechanical load; several of these have been shown to be hyposialylated in GNE myopathy [8–12, 35]. Reduced α2,6 sialylation has been shown to cause TNFR1 activation [36], which in muscle is associated with an oxidative response, decreased muscle strength, and transcription of atrogenes leading to muscle atrophy [37, 38]. Although some atrogenes are upregulated in a mouse model of GNE myopathy [39], this has not been investigated in patients. More studies are needed to understand the role of hyposialylation in the mechanisms leading to muscle atrophy in GNE myopathy. Taken together, the increase in plasma Neu5Ac, intracellular CMP-Neu5Ac, and sarcolemmal sialylation provide robust evidence that ManNAc addresses the underlying biochemical defect at the target tissue in patients with GNE myopathy. Given this mechanistic effect, ManNAc could be evaluated as therapeutic option in other hyposialylation disorders.

Regarding clinical efficacy, we evaluated whether ManNAc would slow or stop the rate of disease progression, since GNE myopathy is a progressive disease leading to skeletal muscle atrophy. Despite the small sample size, we observed a decreased rate of decline in quantitative measures of upper and lower extremity strength and in the AMAT, a reliable measure of physical function, in patients treated with ManNAc compared to natural history [29]. This was not observed when evaluating the 6MWT or PROs, which was not surprising given the lower sensitivity of these measures in GNE myopathy. One caveat to the interpretation of clinical efficacy results is that the baseline characteristics, such as genotype and disease age of the participants in this study were not matched to those of the natural history cohort.

Finally, we tested the performance of the GNE Myopathy Disease Progression Model (GNE-DPM) to determine a treatment effect. This Bayesian model based on quantitative muscle strength was developed to determine disease progression and reduce the number of subjects required to power clinical trials for GNE myopathy [29]. The evaluation of treatment effect using the GNE-DPM showed a reduction of 39% (γ = 0.61) and 45% (γ = 0.55) in disease progression at 12 and 18 months, respectively, but had inconsistent results at 24 and 30 months. Some of the factors considered for the inconsistent GNE-DPM treatment effects estimates included patient dropout, including loss of high responders, the unnoticed dysfunction of the grip dynamometer during the 24- and 30-month visits leading to unreliable data from this muscle used for the GNE-DPM estimates, decreased treatment compliance, and limited durability of the treatment effect.

In summary, oral administration of ManNAc showed long-term safety and manageable tolerability, biochemical efficacy consistent with the intended mechanism of action in skeletal muscle, and preliminary evidence of clinical efficacy. These results informed the design of a multicenter, randomized, double-blind, placebo-controlled study to evaluate the clinical efficacy of ManNAc in GNE myopathy (ClinicalTrials.gov NCT04231266). More broadly, the mechanistic effect of ManNAc provides proof-of-principle evidence for its use in the treatment of hyposialylation disorders and illustrates the potential therapeutic use of drugs that target glycosylation [40].

## Supplementary information

Supplemental Materials

## Data Availability

Anonymized, individual and trial-level data (analysis data sets), and other information (e.g., protocols and clinical study reports) will be shared upon request and after execution of a mutually acceptable Data Sharing Agreement as long as the data is not part of an ongoing or planned regulatory submission. The Data Sharing Agreement will include a restriction that shared unpublished information and data from this study cannot be used by the data recipient for regulatory purposes related to N-acetylmannosamine “ManNAc” as a therapeutic drug until after ManNAc has been reviewed by the regulatory authorities under a New Drug Application as a treatment for GNE myopathy. All such inquiries should be directed to the corresponding author.
