# Supplementary material for: Safety and efficacy of N-acetylmannosamine (ManNAc) in patients with GNE myopathy: an open-label phase 2 study
Source: Genet Med. 2021 Jul 13;23(11):2067–75. doi: 10.1038/s41436-021-01259-x (PMC8553608; doi:10.1038/s41436-021-01259-x)
Supplement: Supplementary file 1 — Supplemental Materials [file 41436_2021_1259_MOESM1_ESM.pdf]

Supplemental Table 1. Treatment-emergent AEs

	**Events (n)**	**Patients (n, %)**
Any serious adverse event	0	-
Any adverse event	196	12 (100%)
Any gastrointestinal adverse event	25	10 (83.3%)
Bloating	5	5 (41.7%)
Diarrhea	5	3 (25.0%)
Flatulence	11	8 (66.7%)
Anemia	8	3 (25.0%)
Fall	6	5 (41.7%)
ALT increased	18	8 (66.7%)
AST increased	7	6 (50.0%)
Bilirubin increased	5	1 (8.3%)
CPK increased	20	10 (83.3%)
Cholesterol high	4	3 (25.0%)
GGT increased	3	3 (25%)
Weight gain	10	5 (41.7%)
aPPT increased	19	7 (58.3%)
Hyperglycemia	9	6 (50.0%)
Hypertriglyceridemia	9	4 (33.3%)
Hypoalbuminemia	5	1 (8.3%)
Headache	5	3 (25.0%)
Rash maculo-papular	3	2 (16.7%)

Adverse events that occurred after initiation of treatment, with a frequency of more than 20% of patients (i.e. occurring in more than 2 patients).

Supplemental Table 2. Pharmacokinetic parameters for plasma ManNAc and Neu5Ac

			Plasma ManNAc[a]				Plasma Neu5Ac[b]			
**Study Day**	**ManNAc dose**	**n**	**T_{max} (h)**	**C_{max}[1] (ng/ml)**	**AUC_{last}[2] (hr*ng/ml)**	**$t_{1/2}$[3] (h)**	**T_{max} (h)**	**C_{max}[1] (ng/ml)**	**AUC_{last}[2] (hr*ng/ml)**	**$t_{1/2}$[3] (h)**
Day 1	3 g BID	6	2.0	1356	7438	3.2	6.0	258	2506	14
Day 1	6 g BID	6	3.0	1672	10492	3.5	9.0	259	2311	*
Day 7	3 g BID	6	2.0	1679	8511	3.2	8.0	632	5969	26
Day 7	6 g BID	6	2.5	1869	10506	2.8	6.0	745	6649	28
Day 90	6 g BID	12	2.0	2152	11443	2.7	8.0	802	7813	11
30 months	4 g TID	8	2.0	1631	31091	*	24	549	18,638	*

Pharmacokinetic parameter calculations conducted without baseline adjustment. SI conversion for [a]Plasma ManNAc (ng/ml)*4.517=nmol/L, and [b]Plasma Neu5Ac (ng/ml)*3.237=nmol/L. Data are [1]Geometric mean, [2]median, [3]mean. *Unable to calculate. Dose escalated to 6 g BID (12 g/day) after Day 7. ManNAc intake stopped for 5-7 days before PK evaluations of a 4 g TID at the 30-month visit. **Abbreviations**: AUClast=area under the plasma concentration-versus time curve from time 0 (dosing) to the time of last quantifiable concentration. BID=twice daily. Cmax=maximum observed plasma concentration. t½=apparent terminal-phase half-life. TID=three times daily. Tmax=time to maximum observed plasma concentration.

Supplemental Table 3. Rate of decline for various clinical outcome measures

	**Natural History annual change Mean (SD) *Quintana et al.***	**ManNAc phase 2 annual change Mean (SD)**	**P-value**
Upper extremity strength (kg)	-4.52 (10.2)	0.21 (9.86)	0.0139
Lower extremity strength (kg)	-9.55 (20.6)	-0.38 (18.83)	0.0006
6MWT (meters)	-10.70 (32.7)	-10.81 (47.67)	NA
AMAT total score	-1.47 (2.52)	-0.83 (2.07)	0.0453
AMAT endurance score	-0.53 (1.41)	-0.47 (1.31)	0.7365
AMAT functional score	-0.90 (1.69)	-0.36 (1.13)	0.0079
HAP adjusted activity score	-1.11 (5.52)	-2.86 (5.53)	NA
HAP maximum activity score	-3.24 (7.96)	-1.44 (7.65)	0.1337
IBMFRS Total Score	-0.49 (1.65)	-1.59 (2.46)	NA

The estimated annual change for various clinical outcome measures estimated by fitting a linear model in the change from baseline as a function of the length of follow-up in patients treated with ManNAc compared to our previously published natural history estimates (*Quintana et al. 2019*) using least squares regression to estimate whether the rate of decline slowed significantly (P-value) compared to natural history.

6MWT=6-minute walk test. AMAT=Adult Myopathy Assessment Tool. HAP=Human Activity Profile. IBMFRS=Inclusion Body Myositis Functional Rating Scale. NA=not applicable.

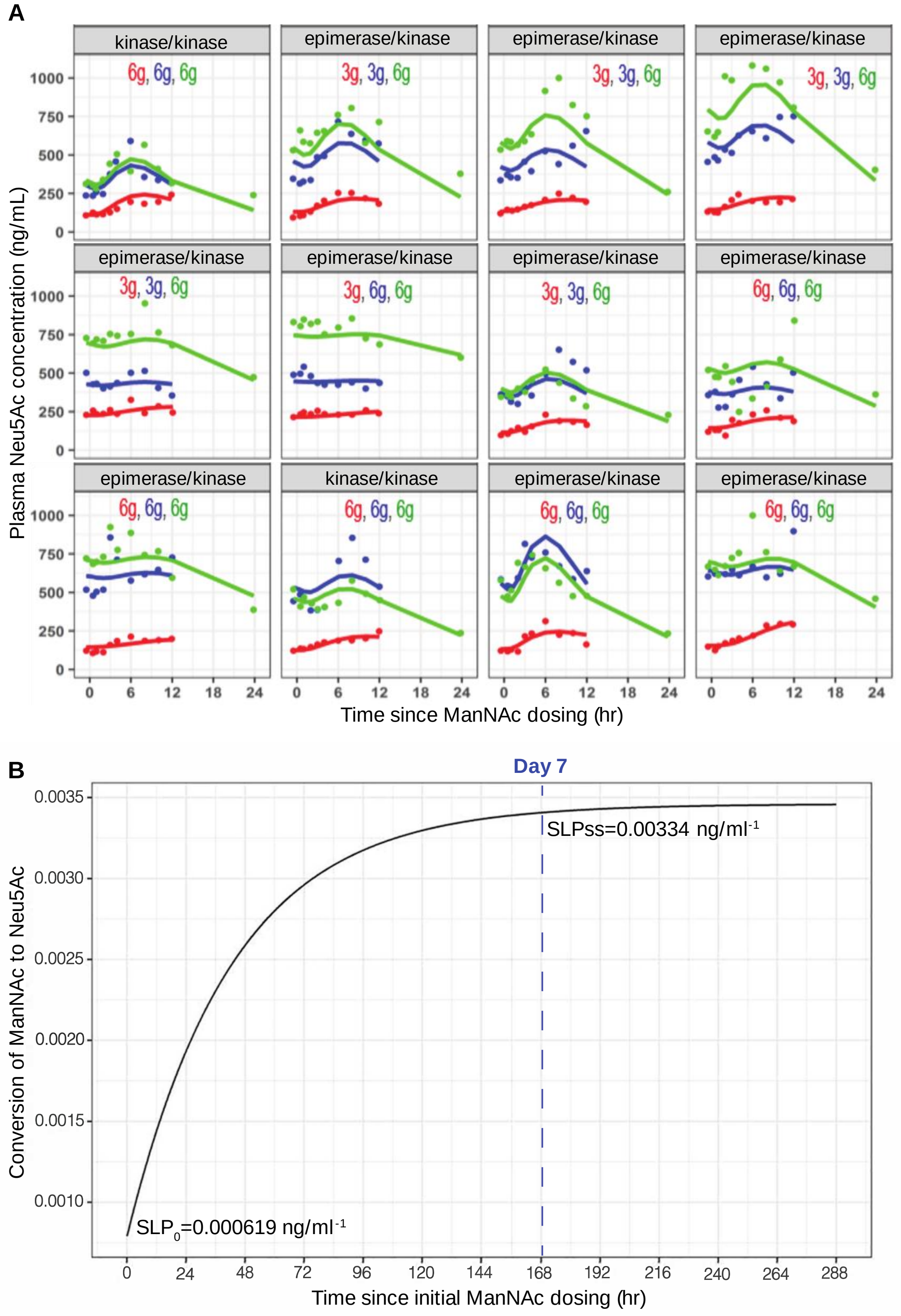

A
kinase/kinase
epimerase/kinase
6g, 6g, 6g
3g, 3g, 6g
3g, 6g, 6g
Plasma Neu5Ac concentration (ng/mL)
Time since ManNAc dosing (hr)
B
Day 7
SLPss=0.00334 ng/ml-1
SLP0=0.000619 ng/ml-1
Conversion of ManNAc to Neu5Ac
Time since initial ManNAc dosing (hr)

Supplemental Fig. 1. (**A**) Individual post-hoc predicted profiles for plasma Neu5Ac concentrations using the population PK/PD model overlaid upon the observed (symbols) concentration-time data on Day 1 (*red*), Day 7 (*blue*) and Day 90 (*green*) at ManNAc doses of 3 g BID or 6 g BID. The *GNE* domains (epimerase or kinase) affected in each patient are indicated. SI conversion for plasma Neu5Ac (ng/ml)*3.237=nmol/L.

(B) Plot of the conversion of ManNAc to Neu5Ac as a function of duration of treatment. The efficiency of Neu5Ac production reaches steady-state (SLPSS) ~7 days after initial dosing.

Abbreviations: BID: twice daily. Neu5Ac: N-acetylneuraminic acid. SLP0: conversion of ManNAc to Neu5Ac at time 0. SLPss: conversion of ManNAc to Neu5Ac at steady-state.
